# *Cryptosporidium* Spp. Infection in Human and Domestic Animals

**Published:** 2012

**Authors:** SM Heidarnegadi, M Mohebali, SH Maraghi, Z Babaei, SH Farnia, A Bairami, M Rezaeian

**Affiliations:** 1 Dept. of Medical Parasitology and Mycology, School of Public Health, Tehran University of Medical Sciences, Iran; 2 Center for Research of Endemic Parasites of Iran (CREPI), Tehran University of Medical Sciences, Tehran, Iran; 3 Dept. of Medical Parasitology and Mycology, School of Medical, Jundishapur University of Medical Sciences, Ahwaz, Iran; 4 Dept. of Medical Parasitology and Mycology, School of Medicine, Kerman University of Medical Sciences, Kerman, Iran

**Keywords:** *Cryptosporidium* infection, Human, Domestic Animal, Iran

## Abstract

**Background:**

*Cryptosporidium* spp. is a coccidian parasite infected humans and animals. Prevalence rate of *Cryptosporidium* spp. infection associated with is some parameters such as sampling, age, season, country and contact to domestic animals. This study aimed to determine *Cryptosporidium* spp. Infection in humans and some animals in rural areas of Shushtar district from Khuzestan Province, south- west of Iran.

**Methods:**

In this study, Stool specimens were randomly collected from 45 cattle, 8 buffalos, 35 calves, 22 turkeys, 3 sheep, 2 geese as well as 62 humans in different seasons selected from rural areas of Shushtar district located in Khuzestan in the south- west of Iran from August 2009 to April 2011. The collected stool samples were examined by modified Ziehl-Neelsen staining method.

**Results:**

Altogether, 68/115 (59.1%) domestic animals and 9/62 (14.5%) of humans were showed *Cryptosporidium* spp. infection in the study areas.

**Conclusion:**

In this study we found the high frequency of *Cryptosporidium* spp. infection in the studied areas.

## Introduction

*Cryptosporidium* is a protozoan parasite that infects the gastrointestinal tract of a wide rang of vertebrates including humans, livestocks, wild animals and birds (1). Cause by covering the extensive host rang, *Cryptosporidium* has been considered to be a zoonotic protozoa (2, 3).*Cryptosporidium* infection can persist for a long time and can lead to serious complications in patients with AIDS (4). But, in patients with an immune system, this organism leads to a self limited infection. Epidemiological studies have publicized that the most important ways of transmission are water born, human-animal and person to person contact (5). *Cryptosporidium* spp. is a main pathogen causing acute diarrhea, non-specific signs including for instance dehydration, fever, anorexia, and weakness may be accompanied. Diarrhea is typically self-limiting in immunocompetent humans. However, it can be major public health importance in children as well as in immunocompromized individuals (6).

This study was designed to investigate the prevalence of *Cryptosporidium* infection in cattle, buffalo, turkey and human especially children exposed with livestock in tropical region of Khuzestan southwestern Iran.

## Materials and Methods

### Fecal sampling

Stool specimens were collected during the different seasons from 45 cattle, 8 buffalo, 53 calves, 22 turkeys, 3 sheep, 2 geese and 62 humans randomly from rural areas of Shushtar City for example Chamtarkhan, Konaarpir, Gelalak and Moraz village in Khuzestan Province, south- west of Iran, between August 2009 and April 2011 (Table 1). For animals, a single fecal sample was collected from the rectum of each animal using disposable plastic bag and transferred to a wide-mouthed disposable plastic container. The specimens were transported to the Intestinal Protozoa Laboratory, School of Public Health, Tehran University of Medical Sciences and preserved in potassium dichromate 5% at 4°C until examined.

**Table 1:** Distribution of *Cryptosporidium spp*. infection in humans

Age (yr)	No. of positive cases	Asymptomatic		Symptomatic	
		No.	%	No.	%
≤10	7	2	22.2	5	55.5
>10	2	1	11.1	1	11.1
Total	9	3	33.3	6	66.6

### Cryptosporidium oocyst detection

Fecal specimens were concentrated by both formol-ether concentration and sheater's flotation (7, 8). Seven ml of the formalin-treated stool specimen and 3 ml of ethyl ether was centrifuged at 650 g for 2 min, resulting in four layers: a layer of ethyl ether, a plug of debris, a layer of formalin-saline, and the sediment. The plug was removed with an applicator stick and the supernatant three layers were decanted. One drop of the sediment poured on to slide and prepared on two microscopically smears were prepared from the sediment and stained by the acid-fast staining. Both of cold and hot method of Modified Ziehl-Neelsen staining was used (9).

### Statistical analysis

Data were analyzed using SPSS (version 13.5; SPSS, Inc, Chicago, IL, USA). A Chi-squared test was used to compare the differences in prevalence of *Cryptosporidium* spp. oocysts between samples of livestock and human with season, age, sex and clinical sign at a 5% level of significance. The prevalence rates were calculated with 95% confidence intervals.

## Results

### Frequency of Cryptosporidium spp.

**Fig. 1:**
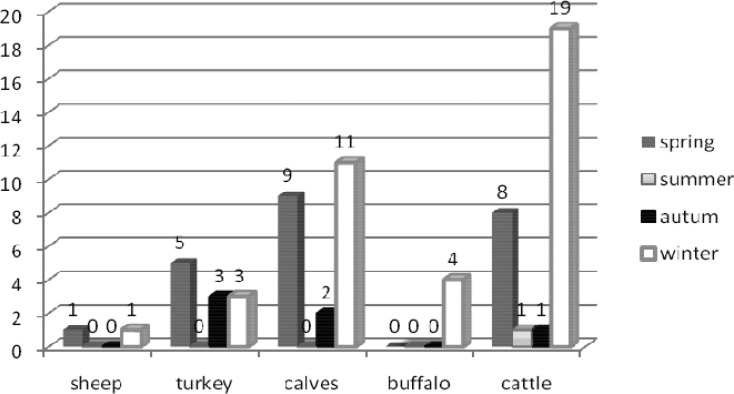
The distribution of *Cryptosporidium* spp. infected among the various animals in different seasons

The overall frequency of *Cryptosporidium* spp. oocysts in animals was 59.1% (68/115) and in human 14.5% (9/62). The highest infection rate of *Cryptosporidium* spp. among animals was 74.5% (38/51) in winter and the lowest in summer 10% (1/10), the infection rate in spring and autumn were 57.5% (23/40), 42.8% (6/14) respectively. The prevalence result of *Cryptosporidium* spp. oocysts among the various animals in different seasons is presented in Fig. 1.

We have found a statistically significant relationship between infection of cryptosporidiosis and season (*P*<0.05) in animals. The infection rate of *Cryptosporidium* spp. in animal at different sampling regions was 81% in Chamtarkhan, 40% in Konaarpir, and 60.9% in Gelalak and Moraz village. The prevalence results of *Cryptosporidium* spp. among human for age and clinical sign are showed in Table 1.

We have not found a statistically significant relationship between infection of cryptosporidiosis and clinical signs and also human sex as well as age. Human contact with animals was 27.7% (10/36) and 12% (3/26) in males and females respectively. Microscopic examination indicated that 40% (6/15) of humans were infected in spring, 7.6% (1/10) in summer, and 23.8% (5/21) in winter. The evaluation of the feces collected showed that infection of *Cryptosporidium* spp. in human in Chamtarkhan was 9.09% (1/11), Gelalak 42.3% (11/26), and Moaraz 4% (1/25).

## Discussion

The prevalence (59.1%) of *Cryptosporidium* spp. in animals obtained in this study was compared with other studies including 6.2% of cattle in Isfahan (10), 35.3% of turkey in the north and west provinces (11), 18.9% of cattle in Kerman (12), and 18.8% of cattle in Qazvin (13). This information indicated that the prevalence of *Cryptosporidium* in this area is higher than in other parts of Iran. Frequency of infection in cattle has been reported from different parts of the world with nearly 40% in Germany, 45.5% incidence in USA, 20% of calves in Canada, 19% of calves in Spain and 27% in Hungary (14, 15–16). The infection rate of *Cryptosporidium* among livestock in some rural parts of Korea was 94% (17).

Our data indicated a potential risk of transmission of *Cryptosporidium* from animal to humans. Mojarad suggested that zoonotic transmission is the main mode of transmission of *Cryptosporidium* infection in Iran (18). One of the most important ways of contamination with *Cryptosporidium* spp. is contact with animals such as cattle, calves and sheep which are important reservoirs of this parasite (19). The prevalence of *Cryptosporidium* among the villagers in several area of Korea was 3.3% (17). Our research indicated that frequency of *Cryptosporidium* spp. among human in these areas was higher than the other rural region of Iran. The prevalence of the parasite in various parts of Iran was 4.1% in west, 7% in southeastern, 2.2% in south, 7.7% in north west, and 2.5% in central parts of Iran (20). We detected that *Cryptosporidium* spp. in children under 10 years was higher than others who were in contact with animals. This finding was confirmed by Joachim et al. (2). *Cryptosporidium* sp. infection has usually been found in children lived close to animals.

The present study indicates no significant difference between the infection rate of *Cryptosporidium* spp. and sex. Males have higher risk than females because they may expose to infections more than females. Our findings also showed that there was no significant difference between the infection rate of *Cryptosporidium* sp. Oocyst and clinical signs.

In winter we had the highest frequency of *Cryptosporidium* spp. in southwest Iran. There was significant association (*P*<.05) between seasons and infection rate of *Cryptosporidium* in animals. Several factors particularly rain may play an important role in formative disseminated of animal to *Cryptosporidium*. The most important factors in *Cryptosporidium* dispreading is rain and the food contamination. Environmental contamination with feces can increase diarrhea in calves and cattle by microorganisms and gastrointestinal parasites (21). In rainy seasons occurrence of *Cryptosporidium* was 6.3% and in dry seasons was 2.7% (22). We detected the highest *Cryptosporidium* spp. in turkeys as 62.5% in spring and the lowest 0% in summer. In some studies, *C. meleagridis* was found in turkey which signifies the great risk of infection due to closer contact of livestock with people who live enclose proximity to them. Evoy et al. described *C. meleagridis* an ‘ avian species’ as a significant human pathogen suggested that turkey may play an important role in zoonotic *Cryptosporidium* transmission (23). The first infection of *C.meleagridis* in turkeys in Iran was reported by Meamar (24). In our studies we also found presence of *Cryptosporidium* oocyst in buffaloes. *Cryptosporidium* spp. have been detected in the feces of buffalos in Italy, Egypt, Cuba, India and Brazil (25).

Important factors in dissemination of the parasite are weather, location of sampling, infection dose and diversity of animal in area. Our study indicated that frequency of *Cryptosporidium* spp. infection in these areas was higher than the other rural regions of Iran and also animals could be an important source of infection in human.
